# The Reliability of Computer-Assisted Three-Dimensional Surgical Simulation of Posterior Osteotomies in Thoracolumbar Kyphosis Secondary to Ankylosing Spondylitis Patients

**DOI:** 10.1155/2022/8134242

**Published:** 2022-08-29

**Authors:** Yiqi Zhang, Yong Hai, Yuzeng Liu, Xinuo Zhang, Yangpu Zhang, Chaofan Han, Jingwei Liu, Lijin Zhou

**Affiliations:** ^1^Department of Orthopedics, Beijing Chaoyang Hospital, Capital Medical University, Gongtinanlu 8#, Chaoyang District, Beijing 100020, China; ^2^Department of Orthopedics, Beijing Hospital, Peking University, DongdandahuaLu 1#, Dongcheng District, Beijing 100005, China

## Abstract

**Objectives:**

The study was aimed at investigating the reliability of computer-assisted three-dimensional surgical simulation (CA3DSS) of posterior osteotomies in thoracolumbar kyphosis secondary to ankylosing spondylitis (TLKAS) patients.

**Methods:**

Eligible TLKAS patients who underwent posterior correction surgery with posterior osteotomies were consecutively included. Simulated posterior osteotomies were performed in Mimics and 3-Matic Medical software. Coronal and sagittal angle and alignment parameters were measured in preoperative full-length X-ray, preoperative original 3D spine (Pre-OS), simulated 3D spine (SS), and postoperative original 3D spine (Post-OS). Reliability was tested by both intraclass correlation coefficients (ICCs) and Bland-Altman analysis.

**Results:**

A total of 30 TLKAS patients were included. Excellent consistency of radiological parameters was shown between preoperative X-ray and Pre-OS model. In SS and Post-OS models, excellent reliabilities were shown in global kyphosis (ICC 0.832, 95% CI 0.677-0.916), thoracic kyphosis (ICC 0.773, 95% CI 0.577-0.885), and lumbar lordosis (ICC 0.896, 95% CI 0.794-0.949) and good reliabilities were exhibited in the main curve (ICC 0.680, 95% CI 0.428-0.834) and sagittal vertical axis (ICC 0.619, 95% CI 0.338-0.798). ICCs of correction angle achieved by pedicle subtraction osteotomy (PSO) was 0.754 (95% CI 0.487-0.892), and that of posterior column osteotomies (PCO) was 0.703 (95% CI 0.511-0.829). Bland-Altman analysis also showed good agreement for both Cobb angle and distance measurements in Pre-OS and SS models, and good reliabilities were shown in PCO and PSO in real spine and SS models.

**Conclusions:**

CA3DSS can provide an accurate measurement, and it is a reliable and effective method to conduct proper simulation for correction surgery with posterior osteotomies in TLKAS patients. This trial is registered with Chinese Clinical Trial Registry ChiCTR2100053808.

## 1. Introduction

Thoracolumbar kyphosis is commonly caused by untreated ankylosing spondylitis (AS), which is a chronic inflammatory disease involving ankylosis of the sacroiliac joint and ossification of the spinal ligament and joint [1–3]. Besides the kyphotic deformity, AS may lead to spinal pseudarthrosis on account of trauma, delayed ossification, severe pain, and neurologic symptoms caused by fibro-osseous tissue progress around the lesion [4, 5]. Thus, correction surgery is desired with the aim to restore the normal sagittal balance and generally performed with posterior osteotomies including pedicle subtraction osteotomy (PSO), Smith-Petersen osteotomy (SPO), and Ponte osteotomy [6, 7].

Both posterior column osteotomies (PCO) and 3-column osteotomies (3CO) are widely applied in kyphotic deformity correction and can achieve adequate and satisfying outcomes in the aspect of radiography and cosmetic [8, 9], nevertheless, the risk of perioperative complications could not be ignored and might be catastrophic in certain circumstance [10, 11]. Therefore, it is essential to make a meticulous preoperative surgical plan for osteotomy in correction surgery.

Several studies have reported 2D correction simulation to predict designed osteotomy plan based on sagittal or coronal X-ray, and the feasibility of 2D simulation in PSO has been reported [12, 13]. Although 2D simulation provided a reference about osteotomy location based on X-ray, most spinal deformities are 3D malformation of the spine. Thus, it might be more essential to design osteotomy plan with more anatomical details in 3D view to avoid iatrogenic injury. Multilevel PCO is an important correction technique for TLKAS patients, but 2D simulation was scarcely performed in multilevel PCO which may be due to the faint identification of posterior elements in X-ray.

Recently, researches have confirmed the application of 3D reconstruction technique with Mimics Medical software in hip trauma, orthopedic oncology, and cervical spine surgery for parameter measurement and 3D printing technology [14–16]. 3D simulation better allows the surgeon to improve the visualization of the patient's anatomy and perform the procedures through virtual omnidirectional feedback. The technique could compensate the deficiency of 2D simulation caused by faint identification of posterior elements [17]. Therefore, we presumed that it is more beneficial for TLKAS patients to accept 3D simulation, especially for patients who plan to undergo multilevel PCO. Nevertheless, the reliability of 3D simulation in posterior osteotomy simulation for TLKAS remains unknown yet. Given the paucity of the data in this field, we intended to initially explore the Mimics Medical and related software to provide an accurate, flexible, and intuitive 3D simulation to make surgical plan for posterior osteotomies based on CT scan data and aimed to investigate the reliability of CA3DSS for posterior osteotomies in TLKAS.

## 2. Materials and Methods

### 2.1. Patients

Eligible TLKAS patients were consecutively included in the study from January 2017 to November 2021. Inclusion criteria were as follows: (1) patients were diagnosed as AS by laboratory tests, radiological features, and clinical manifestations guided by the New York criteria [18] and (2) all the patients received one-stage posterior correction surgery due to kyphosis with pedicle screw fixation and posterior osteotomies at thoracolumbar spine by the same surgeon in the institution. Exclusion criteria were (1) patients with incomplete clinical or imaging data; (2) AS patients progressed kyphosis because of trauma, infection, and tumor; and (3) the patient had a history of spinal surgery. The study was approved by the institutional review board of our institution.

### 2.2. 3D Spine Model Reconstruction

Patients' CT scan data of the whole spine were collected with Digital Imaging and Communications in Medicine (DICOM) format (DICOM format data from Siemens CT machine, SOMATOM Sensation 16, Siemens AG, Forchheim, Germany). All the tomographic pictures were imported into Mimics Medical 21.0 (Materialise NV, Leuven, Belgium), and 3D spine model was established with threshold of 226-3071HU. Further parameter measurement and surgical planning were calculated and simulated in 3-Matic Medical 13.0 (Materialise NV, Leuven, Belgium) after importing the created 3D model.

**Figure 1:**
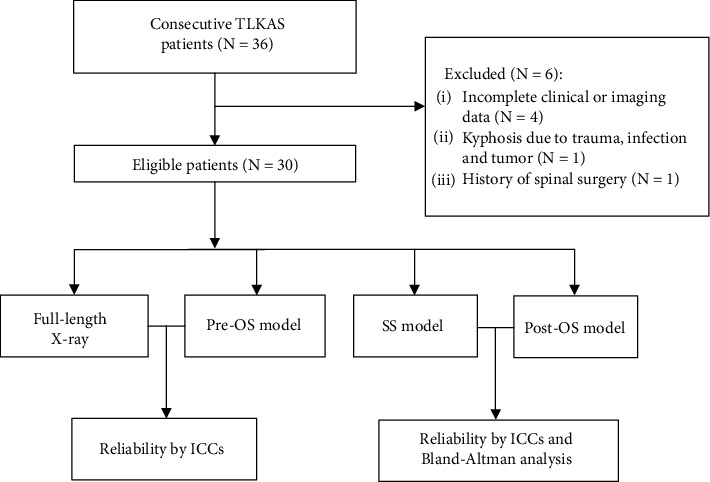
The flowchart. TLKAS: thoracolumbar kyphosis secondary to ankylosing spondylitis; Pre-OS: preoperative original 3D spine; SS: simulated 3D spine; Post-OS: postoperative original 3D spine.

Patients' radiological data were collected and analyzed. The measurement consistency was evaluated between X-ray and preoperative original 3D spine (Pre-OS) models. The results of osteotomy simulation by CA3DSS were evaluated by the measurement in simulated 3D spine (SS) and postoperative original 3D spine (Post-OS) models. The reliability of angle change by different posterior osteotomies was also assessed. Pre-OS model was the 3D spine model reconstructed with preoperative CT scan data, and SS model was the one that Pre-OS model underwent osteotomy simulation. Post-OS model was reconstructed 3D spine model with postoperative CT scan data. The flowchart is depicted in Figure 1.

### 2.3. Parameter Measurement

For 2D radiological measurement, preoperative full-length anteroposterior and lateral spine X-ray were collected. Coronal and sagittal parameters were documented including global kyphosis (GK), thoracic kyphosis (TK), lumbar lordosis (LL), sagittal vertical axis (SVA), and main curve (MC). GK was defined as the largest Cobb angle in sagittal plane, and MC was defined as the Cobb angle of the main curve in coronal plane.

**Figure 2:**
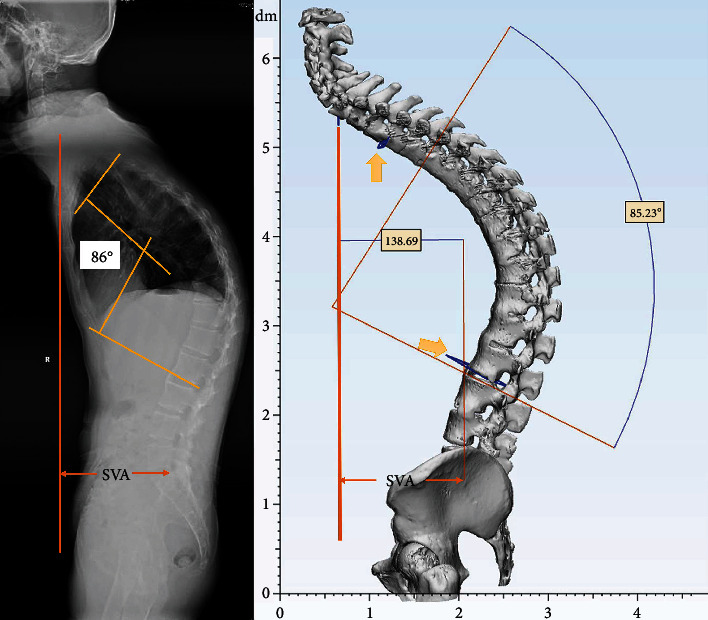
Measurements in Pre-OS. Yellow arrows indicated that planes paralleling to the end plates were used for Cobb angle measurement.

For 3D radiological measurement, the mentioned parameters were documented with measuring tools in 3-Matic Medical 13.0 in Pre-OS, SS, and Post-OS models. To maintain an accurate data, the 3D spine model was initially adjusted to the same position as full-length AP view X-ray according to the pelvic position in sagittal plane. The Cobb angle was achieved by angle measurement with two planes paralleling to upper and lower end plate according to the end vertebras settled in X-ray. SVA was measured by the vertical distance from posterior upper margin of S1 to the vertical plane settled by middle point of C7 (Figure 2).

### 2.4. Posterior Osteotomy Simulation

**Figure 3:**
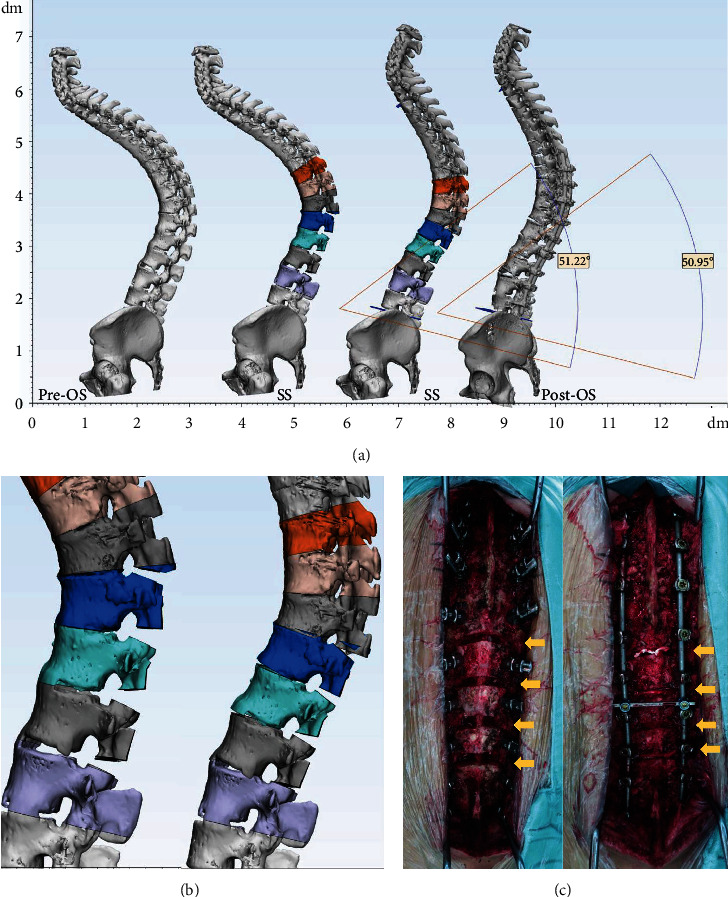
3D simulation of multilevel PCO. (a) Sagittal view of the 3D spine models. (b) Partial view of PCO simulation. (c) Intra-op photographs. Yellow arrows indicated osteotomy parts.

**Figure 4:**
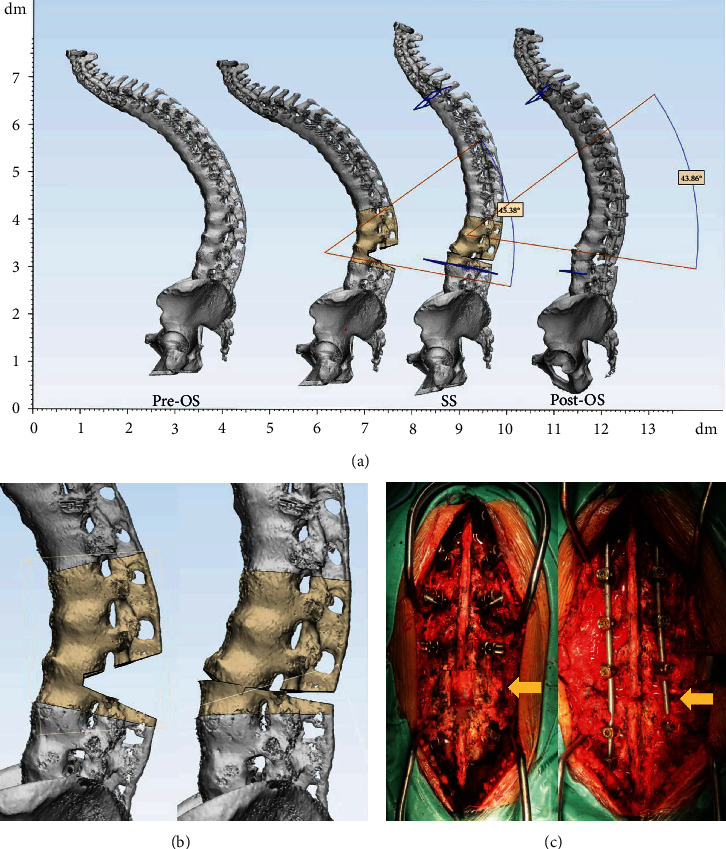
3D simulation of PSO. (a) Sagittal view of the 3D spine models. (b) Partial view of PSO simulation. (c) Intra-op photographs and yellow arrows indicated L3 PSO.

**Figure 5:**
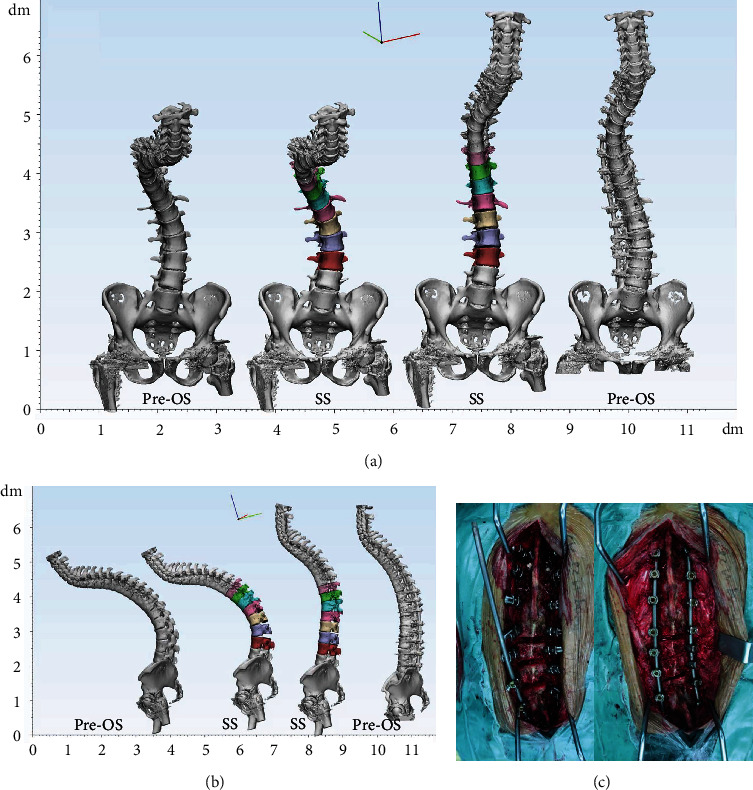
3D simulation of multilevel PCO for TLKAS patient with scoliosis. (a) Coronal view of the 3D spine models. (b) Sagittal view of the 3D spine models. (c) Intra-op photographs of multilevel PCO and yellow arrows indicated PCO at T12/L1, L1/2, and L2/3.

Posterior osteotomy simulation was performed in 3-Matic Medical 13.0 with cut tool and trim tool in Pre-OS model based on intra-op pictures and operation records. During the simulation process, the operators were blinded to Post-OS model. After trimming the targeted posterior element, the rotation tool was used to achieve the wedge closing process. PCO and PSO simulations were accomplished following the literatures [7, 8] and surgeon's experience (Figures 3 and 4). For patients with coronal curve, coronal correction simulation was achieved after sagittal procedure (Figure 5). The osteotomy angle was assessed in Pre-OS, SS, and Post-OS models by angle calculation according to the angle change of upper and lower end plate in targeted vertebras.

Both 2D and 3D radiological measurements were performed by two experienced surgeons, and any discrepancy was solved by reevaluation and discussion between them. The reliability of CA3DSS was assessed by global measurement and local measurement. Global measurement included GK, TK, LL, MC, and SVA. Local measurement included the angle change of different posterior osteotomies.

### 2.5. Statistics Analysis

Quantitative data were listed as means ± SD or as medians with interquartile range when the data presented a nonnormal distribution. The reliability of the parameters documented in X-ray, Pre-OS, SS, and Post-OS models were determined by intraclass correlation coefficients (ICCs). Reliabilities below 0.40 were considered as poor, 0.40 to 0.75 were fair to good, and >0.75 were characterized as excellent. All statistical analysis were calculated by SPSS Statistics 20 (IBM Corp, Armonk, New York, United States). Bias of the data conforming to normal distribution were analyzed using Bland-Altman analysis to evaluate the agreement between the mentioned corresponding parameters by GraphPad Prism 6 (GraphPad Software, La Jolla, CA).

## 3. Results

After screening 36 patients, a total of 30 eligible TLKAS patients were finally included in the study. All patients underwent posterior pedicle screw fixation and posterior osteotomies for correction. The average age was 40 ± 5.8 years old. One-level PSO osteotomy was performed in 11 patients and two-level PSO osteotomy in 2 patients. Multi-PCO and hybrid osteotomy (PSO + multi − PCO) were performed in 11 patients and 6 patients, respectively.

### 3.1. Consistency between 2D and 3D Measurements

For preoperative measurement in both full-length X-ray and Pre-OS, excellent reliabilities were exhibited in GK (ICC 0.942, 95% CI 0.882-0.972), TK (ICC 0.954, 95% CI 0.907-0.978), LL (ICC 0.955, 95% CI 0.908-0.978), MC (ICC 0.992, 95% CI 0.983-0.996), and SVA (ICC 0.965, 95% CI 0.928-0.983) (Table 1).

### 3.2. Reliability of 3D Simulation by Global Measurement

**Figure 6:**
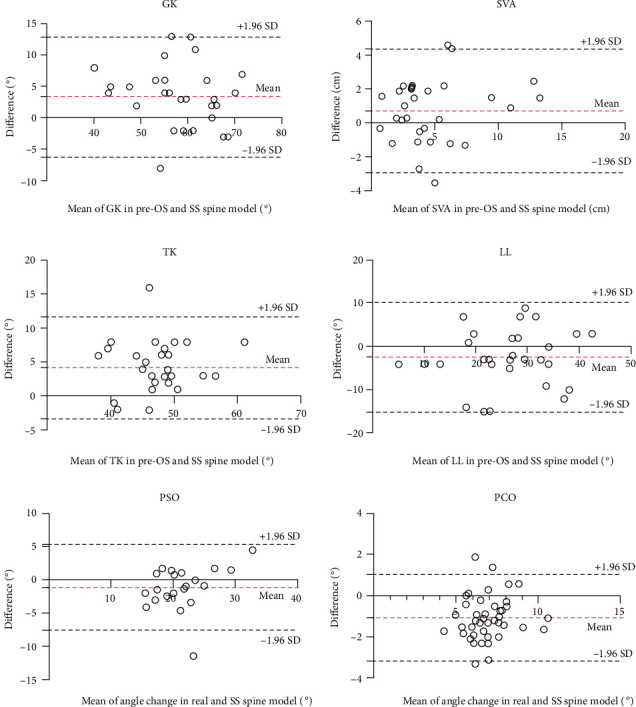
Bland-Altman analysis of radiological parameters. Red dashed lines indicated the mean difference with the corresponding 95% CI, and black dashed lines indicated the limits of agreement with the corresponding 95% CI.

For postoperative radiological parameters between Post-OS and SS models, excellent reliabilities were exhibited in GK (ICC 0.832, 95% CI 0.677-0.916), TK (ICC 0.773, 95% CI 0.577-0.885), and LL (ICC 0.896, 95% CI 0.794-0.949) and good reliabilities were shown in MC (ICC 0.680, 95% CI 0.428-0.834) and SVA (ICC 0.619, 95% CI 0.338-0.798) (Table 2). Bland-Altman analysis indicated acceptable agreement for GK, TK, LL, and SVA in SS and Post-OS models (Figure 6).

### 3.3. Reliability of 3D Simulation by Local Measurement

For measurement of posterior osteotomies, PCO showed 6.39° ± 1.45° for each level in real spine, and the consistency was good with an ICC value of 0.703 (95% CI 0.511-0.829). PSO procedure showed 20.84° ± 5.16° correction in each vertebra and achieved an excellent reliability (ICC 0.754, 95% CI 0.487-0.892) (Table 3). Bland-Altman analysis indicated good reliabilities for PCO and PSO in real spine and SS models (Figure 6).

## 4. Discussion

Approximately 30% of the AS patients will develop into thoracolumbar kyphosis [1]. Generally, the surgical target is to restore sagittal balance from flexed trunk with posterior osteotomies including PSO and PCO. Posterior osteotomies have been widely used in spinal deformity correction surgery and can provide an adequate correction and clinical outcomes especially for patients with kyphotic deformity [5–7, 19]. Whereas complications associated with posterior osteotomies should not be ignored, literatures have disclosed that the total complication rates were 43% and 28% for SPO and PSO, respectively, and the rates of neurological deficits were 6% for SPO and 5% for PSO [20]. To ensure patients to achieve an accurate, safe, and sufficient correction, computer-assisted surgical planning has been widely used in the application of preoperative osteotomy planning for AS patients [13, 21, 22]. Zhang et al. [21] have reported the application of Surgimap software in two-level PSO in kyphosis after AS and considered it was a feasible, safe, and effective method for individual treatment. Langella et al. [23] demonstrated the ability of Surgimap in prediction of proper alignment for sagittal imbalance. Previous studies have reported single-level posterior osteotomy simulation, and few have paid attention to multilevel PCO, which would be difficult with 2D simulation due to faint identification of posterior elements in X-ray. The simulation based on full-length X-ray could also bring difficulty in identifying where or how to perform the osteotomy procedure in detail [13, 24]. In addition, spinal deformities are 3D malformation of the spine, and in clinical work, we found that both sagittal imbalance and coronal imbalance could impact AS patients. 2D simulation could not show coronal and sagittal views in the same time. Hence, a real 3D and simulation is demanded.

In the present study, we have explored the reliability of Mimics and related software in TLKAS. The results showed proper predictive abilities in both angle and alignment. Considering the stiffness of spinal change in AS, we thought there might be no much difference in standing and supine radiographs, compared to a deviation of 8.8° magnitude in idiopathic scoliosis [25]. On the comparison of measurement, we noticed that there was a 1-3° magnitude between X-ray and 3D spine in Cobb angle, which just verified our mentioned assumption. And this also verified the feasibility of angle and alignment measurements in 3D spine model. The results also demonstrated an excellent consistency between SS and Post-OS models in sagittal parameters. The coronal parameter of MC showed slightly lower ICC value, but it was still in the reliable reference. Due to the abnormal distribution of the data, we did not perform Bland-Altman analysis of MC. Most of the simulated osteotomies were performed firstly in the sagittal plane, and then, coronal closing process would be performed which would affect the accuracy of coronal parameters, which might cause the lower ICC value of MC. In the cohort, the results of SS model showed 2°-4° deviation of GK compared to Post-OS, and we thought that the deviation occurred in osteotomy closing process. In PSO procedure, a 25°-35° correction of lordosis would be achieved though vertebral body wedge shorten after posterior element removal [8, 26, 27]. In the present study, we demonstrated a 20.8° correction for PSO procedure, and ICCs showed good agreement in SS model as 21.9°. For PCO procedure, literatures demonstrated a 5°-10° correction achieved with posterior element resection and gap closings, and multilevel PCO could acquire outstanding and safe outcomes [3, 7, 28, 29]. The efficacy of PCO in our study showed a 6.39° correction in each level and 7.45° as a result of simulation. The consistency was marginally worse compared to PSO procedure, and the reason we thought were as listed. First, PCO theoretically achieved a 5°-10° angle change while we could not ignore the measure error for 1°-3° as researches reported [30, 31]. Second, we found hardly that the closure of posterior elements could be matched exactly in real closing process, a desired closure could not be achieved in real although we simulated a relatively matched closure, and this also occurred in PSO simulation. However, a multilevel PCO tended to magnify the margin, and thus, a marginally worse result was indicated compared to PSO simulation. Despite the reliability of simulated PCO was not superior compared to simulated PSO, both of the simulated osteotomies demonstrated good predictability.

Computer-assisted virtual surgical planning has been widely used in 3D measurement and 3D printing technique [16, 32, 33]. The technique allows the surgeon to visualize the patient's anatomy thoroughly. In the present study, we demonstrated a 3D visual, flexible, and highly consistent simulation with the software for posterior osteotomies in TLKAS patients. This technique can provide intuitive reference and comprehension of posterior osteotomy, and we believed the technique can bring surgeons anatomy measurement and correction design preoperatively and further studies may be applied in other spinal deformities.

The limitations of the present study include that it was a retrospective study and the sample size was small. The simulation was calculated based on preoperative and postoperative supine position CT data, and SVA bias may exist in patients with mobilizable hip joint in upright position after its compensation compared to postoperative stand X-ray. This limitation exists in all the 2D and 3D simulation software, which is hard to overcome at present. So far, we believed this study can provide a great reference for further research.

## 5. Conclusions

The results of radiological measurements with CA3DSS are almost the same compared to 2D measurement. CA3DSS is reliable for posterior osteotomy simulation in TLKAS patients. The application of the technique is effective and would help surgeons verify the osteotomy location preoperatively and evaluate the magnitude for osteotomy procedure.

## Figures and Tables

**Table 1:** Results of ICCs for measurement in preoperative full-spine X-ray and Pre-OS models.

Parameters	X-ray	Pre-OS	ICC (95% CI)
GK (°)	87.86 ± 13.25	85.36 ± 13.14	0.942 (0.882-0.972)
TK (°)	65.67 ± 11.17	62.7 ± 10.85	0.954 (0.907-0.978)
LL (°)	5.15 ± 11.33	6.64 ± 12.00	0.955 (0.908-0.978)
MC (°)	2.60 (0.75-10.15)	2.55 (0.00-9.00)	0.992 (0.983-0.996)
SVA (cm)	6.95 (5.77-12.73)	7.80 (5.85-12.15)	0.965 (0.928-0.983)

GK: global kyphosis; TK: thoracic kyphosis; LL: lumbar lordosis; MC: main curve; SVA: sagittal vertical axis; Pre-OS: preoperative original 3D spine.

**Table 2:** Results of ICCs and Bland-Altman analysis for radiological measurement in Post-OS and SS models.

Parameters	Post-OS	SS	ICC analysis	Bland-Altman analysis	
			ICC (95% CI)	Mean bias	LOA (95% CI)
GK (°)	60.03 ± 8.02	56.63 ± 8.83	0.832 (0.677-0.916)	3.400	-6.183-12.98
TK (°)	49.70 ± 5.55	45.47 ± 5.27	0.773 (0.577-0.885)	4.233	-3.256-11.72
LL (°)	25.39 ± 10.05	27.73 ± 9.12	0.896 (0.794-0.949)	-0.2330	-15.12-10.46
MC	0.00 (0.50-1.25)	0 (0.00-0.00)	0.680 (0.428-0.834)	—	—
SVA (cm)	4.10 (3.05-6.72)	3.85 (2.10-5.58)	0.619 (0.338-0.798)	0.727	-2.902-4.356

MC and SVA were exhibited as medians with interquartile range. SS: simulated 3D spine; Post-OS: postoperative original 3D spine.

**Table 3:** Results of ICCs and Bland-Altman analysis for the angle changes in real and simulated osteotomies.

Type of osteotomy	Real spine	Simulation	ICC analysis	Bland-Altman analysis	
			ICC (95% CI)	Mean bias	LOA (95% CI)
PCO (°, *n* = 42)	6.39 ± 1.45	7.45 ± 1.33	0.703 (0.511-0.829)	-1.062	-3.167-1.043
PSO (°, *n* = 21)	20.84 ± 5.16	21.90 ± 4.17	0.754 (0.487-0.892)	-1.052	-7.499-5.394

PCO: posterior column osteotomy; PSO: pedicle subtraction osteotomy.

## Data Availability

The datasets used and/or analyzed during the current study are not publicly available due to the data being confidential; however, they are available from the corresponding author on a reasonable request.
